# Gasotransmitters in the regulation of connexins in central and peripheral nervous system injuries

**DOI:** 10.4103/mgr.MEDGASRES-D-25-00156

**Published:** 2026-05-21

**Authors:** Stanislav Rodkin

**Affiliations:** Research Laboratory “Medical Digital Images Based on the Basic Model,” Department of Bioengineering, Faculty of Bioengineering and Veterinary Medicine, Don State Technical University, Rostov-on-Don, Russia

**Keywords:** apoptosis, carbon monoxide, connexin, nitric oxide, gasotransmitter, glial cell, hydrogen sulfide, neuroinflammation, neuron, peripheral nerve injury, spinal cord injury, traumatic brain injury

## Abstract

Gasotransmitters, such as nitric oxide, carbon monoxide, and hydrogen sulfide can freely diffuse across biological membranes, bind to receptors and molecular targets, and modulate multiple intracellular signaling pathways. However, no systematic review exists regarding how gasotransmitters influence central and peripheral nervous system injury by regulating connexins. This review study presents, for the first time, a systematic analysis of the effects of these three gasotransmitters on connexin regulation, with a focus on their potential roles in injuries to the central and peripheral nervous systems. The results of the systematic analysis showed that carbon monoxide can exert both neuroprotective and neurotoxic effects through S-nitrosylation, phosphorylation, and regulation of connexin channels, as well as contribute to neurovascular coupling and ischemic preconditioning. Carbon monoxide primarily modulates connexin activity via the heme oxygenase-1/carbon monoxide/nuclear factor erythroid 2-related factor 2 signaling pathways, demonstrating potential in reducing neuroinflammation and necroptosis. Hydrogen sulfide also affects connexin-related signaling pathways, regulates platelet aggregation and regulatory T cell-mediated immune responses, and supports vascular homeostasis. Importantly, connexins function as molecular hubs integrating gasotransmitter-dependent signals, thereby mediating both secondary injury cascades and neuroregenerative processes. The obtained results indicate that connexins represent promising therapeutic targets, and pharmacological strategies based on gasotransmitters constitute a novel direction in neuroprotection after neural injury. However, the clinical application of these approaches remains limited, and further studies are required to clarify isoform-specific connexin regulation, optimize gasotransmitter delivery systems, and minimize potential systemic toxicity.

## Introduction

Intercellular communication is essential for the functioning of the body, integrating the activity of numerous cells into a coherent system.^1^ However, nervous system injuries cause both local and systemic disruption of cellular connectivity, triggering pathological molecular and cellular events. Developing such treatments requires a deeper understanding of the signaling mechanisms that determine neuronal and glial survival or death following trauma.

Connexins (Cxs), transmembrane proteins that form hemichannels and gap junctions, are key mediators of direct intercellular communication. These structures enable the exchange of ions, metabolites, secondary messengers, and even some organelles.^2^,^3^,^4^ In addition to their classical role as “intercellular bridges,” Cx is involved in cellular proliferation, migration, and survival processes.^5^,^6^,^7^ Neurotraumas are accompanied by dysregulation of Cx expression and post-translational modifications in both neurons and glial cells, contributing to the progression of secondary injuries.^8^,^9^

Gasotransmitters play an important role in the regulation of Cx.^10^,^11^,^12^,^13^,^14^,^15^,^16^,^17^,^18^,^19^,^20^,^21^ These are unique gaseous molecules that can freely diffuse across biological membranes, bind to receptors and molecular targets, and modulate various intracellular signaling pathways.^22^ Nitric oxide (NO), carbon monoxide (CO), and hydrogen sulfide (H_2_S) form the classical triad of gasotransmitters, which perform numerous functions in both physiological and pathological states.^23^,^24^ In the context of neurotrauma, the levels of NO, CO, and H_2_S constitute a dynamic system that plays a key role in determining cell fate through a variety of molecular mechanisms, ranging from the generation and interaction with reactive oxygen species (ROS) to more subtle signaling cascades.^25^,^26^,^27^ However, studies specifically addressing the influence of gasotransmitters on the regulation of Cx in neurotrauma are virtually nonexistent.

Therefore, this study aims to fill the existing knowledge gap regarding gasotransmitter-dependent regulation of Cx in neurotrauma and to lay a theoretical foundation for future experimental and clinical developments in the field of neuroprotection. We conducted a comprehensive and in-depth analysis of the effects of gasotransmitters on Cx regulation in traumatic brain injury (TBI), spinal cord injury (SCI), and peripheral nervous system injuries, identifying key molecular mechanisms underlying Cx transformation and extrapolating gasotransmitter-dependent effects from other pathological models. The gasotransmitter-dependent neuroprotective and cytotoxic effects, mediated through hemichannels and gap junctions, are thoroughly examined.

## Methods

This scoping review was conducted in accordance with the Preferred Reporting Items for Systematic Reviews and Meta-Analyses extension for Scoping Reviews (PRISMA-ScR) guidelines.^28^,^29^ The literature search was conducted using the PubMed, Scopus, Web of Science, and Google Scholar databases, with no restrictions on publication date, language, or full-text availability. Keywords included terms such as “gasotransmitters,” “nitric oxide,” “carbon monoxide,” “hydrogen sulfide,” “connexin,” “neurotrauma,” “traumatic brain injury,” “spinal cord injury,” “peripheral neurotrauma,” and their combinations using Boolean operators.

After the initial screening, the relevance of each publication was assessed based on titles and abstracts. Articles deemed relevant were reviewed in full. Publications with methodological flaws, lack of peer review, or insufficient scientific significance were excluded. The final decision to include a study was made by the author based on an overall assessment of quality and informativeness.

## Structure and Functions of Connexin and Its Gasotransmitter-Dependent Regulation in Nervous Tissue

Cx is a key component of intercellular channels, that enables the exchange of ions, metabolites, and signaling molecules between adjacent cells. Each Cx comprises four transmembrane domains, two extracellular loops, one cytoplasmic loop, and short N-terminal and long C-terminal domains (**Figure 1A**). The extracellular loops mediate the docking of Cx from adjacent cells, while disulfide bridges between conserved cysteine residues stabilize its structure. The cytoplasmic regions regulate channel activity; for instance, the N-terminus is involved in gating mechanisms, and the C-terminus undergoes post-translational modifications, such as phosphorylation, which influence channel assembly, gating, and degradation.^2^,^3^,^4^ It should be noted that the N-terminus represents a voltage-dependent structure involved in gating and is capable of folding into the cytoplasmic pore, thereby blocking its permeability.^30^ In turn, the C-terminus serves as the primary regulator of Cx transport, assembly, and degradation, as well as gating kinetics.^4^ For instance, truncation of the C-terminus alters the kinetics of the voltage-dependent channel, increasing its open time and affecting transitional states.^31^

**Figure 1:**
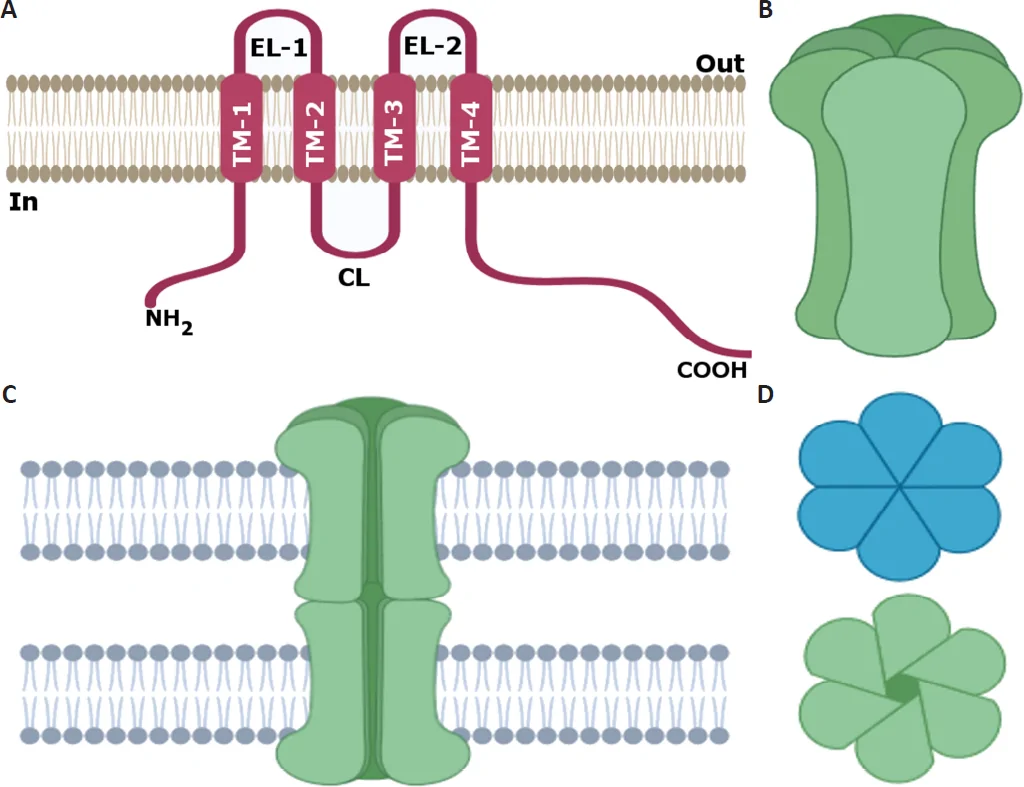
Structure and organization of the Cx43 protein in neuroglial gap junctions. (A) Schematic representation of Cx43 topology. A transmembrane protein with four transmembrane domains (TM1–TM4), two extracellular loops (EL1–EL2), one intracellular loop (CL), and cytoplasmic N (NH_2_)- and C-terminal domains (COOH). (B) Diagram of a hexameric connexon, composed of six Cx subunits. (C) Representation of a gap junction: two hexameric connexons, each formed by six Cx subunits, aligned to create an intercellular channel (shown in green). (D) Illustration of the closed (blue) and open (green) states of a hemichannel, highlighting conformational changes that regulate channel permeability. Created with BioRender.com and Inkscape. Cx: Connexin.

Two hexameric connexons, each composed of six Cx subunits (**Figure 1B**), align across adjacent cell membranes to form a gap junction (**Figure 1C**). The three-dimensional structure of the connexon, including the arrangement of subunits and pore architecture, is shown in ribbon and space-filling representations. Connexons can also function as individual hemichannels that transition between closed and open states depending on conformational changes (**Figure 1D**).^32^,^33^,^34^

The genetic heterogeneity of Cx accounts for the diversity of its functions in different tissues. These functions extend beyond direct molecular transfer via gap junctions and hemichannels and include complex signaling mechanisms that integrate cells into functional ensembles under both physiological and pathological conditions. For example, Cx43 (encoded by the *GJA1* gene), which is widely expressed in the body, forms astrocytic networks in the nervous system that buffer excess K^+^ and glutamate, preventing excitotoxicity.^35^,^36^ These networks also propagate calcium waves and coordinate neurovascular coupling.^37^,^38^ In the peripheral nervous system, Cx32 (encoded by the *GJB1* gene), expressed in Schwann cells and oligodendrocytes, contributes to the metabolic support of myelinated axons, and its mutation can lead to demyelination.^39^,^40^,^41^

In pathological conditions, hemichannels play a critical role. Studies have shown that during inflammation, activation of astrocytic Cx43 hemichannels leads to adenosine triphosphate (ATP) release and activation of microglial purinergic receptors, followed by the expression of pro-inflammatory cytokines, calcium accumulation, and apoptosis.^42^,^43^ However, the role of Cx43 in neurotraumas is highly concentration- and time-dependent, exhibiting dual effects ranging from cytotoxicity to neuroprotection.^44^,^45^ Also, for example, astrocytic Cx43 causes calcium excitotoxicity, oxidative stress, and neuronal homeostasis dysfunction,^44^ and increases edema,^46^ and astrogliosis,^47^ thereby expanding the front of secondary injury in the post-traumatic period. In other cases, Cx43 may exert a neuroprotective effect through the release of exosomes,^48^ act as a molecular bridge for mitochondrial transfer,^49^ and so on.

In neuronal networks, Cx36 (encoded by the GJD2 gene) is of particular importance, as it forms electrical synapses between interneurons.^50^,^51^,^52^ Its expression in the hypothalamus, retina, and cerebellum ensures rapid neuronal synchronization,^53^ and dysfunction is associated with epilepsy and cognitive impairments.^54^,^55^ Additionally, Cx47 (encoded by the *GJC2* gene), expressed in oligodendrocytes, forms gap junctions with astrocytic Cx43, enabling functional integration of glial cells.^56^,^57^ Mutations in Cx47 cause various neuropathological conditions related to white matter damage and corticospinal tract dysfunction.^58^ Cx26 has also been implicated in neurogenesis.^59^

Intercellular communication mediated by Cx is finely regulated by gasotransmitters — gaseous signaling molecules that, despite their simple structure, control complex biological processes. Among them, NO, CO, and H_2_S play pivotal roles in linking cellular metabolism to Cx-dependent signaling pathways.

NO is synthesized from L-arginine by nitric oxide synthases (NOS): endothelial (eNOS), neuronal (nNOS), and inducible (iNOS), which largely determine its dual nature.^60^ While eNOS and nNOS are calcium-dependent and maintain homeostasis, iNOS is induced by stress and produces cytotoxic levels of NO, leading to cell death.^24^,^25^,^61^ NO directly modifies Cx structure via S-nitrosylation of cysteine residues, altering gap junction conductance.^10^,^11^,^14^,^15^,^16^,^17^,^18^ It also induces oxidative damage to Cxs^62^ and regulates several signaling pathways, such as the cyclic guanosine monophosphate (cGMP)/protein kinase A cascade, leading to Cx phosphorylation (**Figure 2**).^63^,^64^

**Figure 2:**
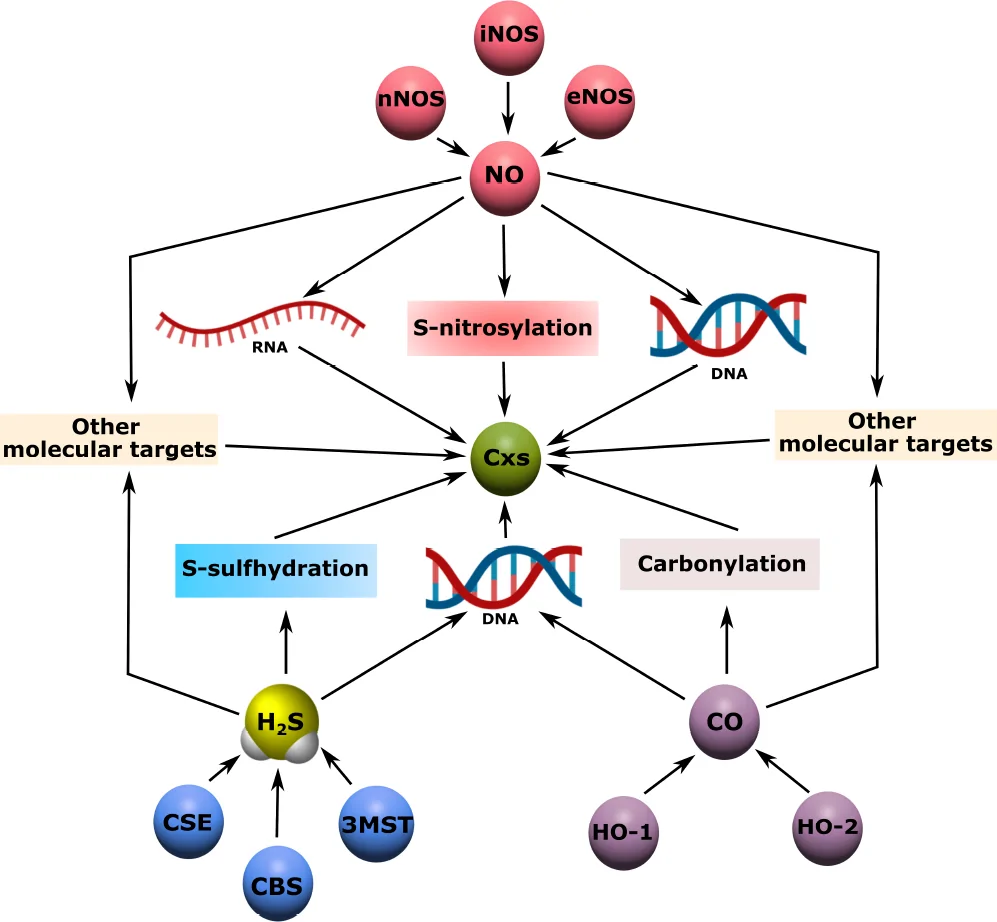
Endogenous synthesis of NO, CO, and H_2_S and their mechanisms of Cx regulation. These gasotransmitters are synthesized by specific enzymes and modulate Cx function via direct modifications such as S-nitrosylation, carbonylation and S-sulfhydration, as well as through indirect signaling cascades that influence Cx expression, trafficking and channel gating. Created with BioRender.com and Inkscape. 3MST: 3-Mercaptopyruvate sulfurtransferase; CBS: cystathionine β-synthase; CO: carbon monoxide; CSE: cystathionine γ-lyase; Cx: connexin; eNOS: endothelial nitric oxide synthase; H_2_S: hydrogen sulfide; HO: heme oxygenase; iNOS: inducible nitric oxide synthase; nNOS: neuronal nitric oxide synthase; NO: nitric oxide.

CO is produced during heme degradation by heme oxygenase-1 (HO-1) and heme oxygenase-2. While heme oxygenase-2 is constitutively active, HO-1 is stress-inducible and increases CO production for cytoprotection.^23^,^24^,^65^ CO can modulate Cx activity via carbonylation, a covalent modification that alters channel function.^19^ Simultaneously, it activates signaling pathways associated with receptor-interacting protein kinase 1, receptor-interacting protein kinase 3, and mixed lineage kinase domain-like protein (MLKL)^66^ and the transcription factor nuclear factor erythroid 2-related factor 2 (Nrf2),^67^ enhancing or suppressing the synthesis of Cx (**Figure 2**).

H_2_S is mainly synthesized by cystathionine β-synthase, cystathionine γ-lyase, and 3-mercaptopyruvate sulfurtransferase,^25^,^68^,^69^ and contributes to the complex regulation of Cx. Several studies suggest that H_2_S modulates Cx expression.^12^,^13^,^20^,^21^ It may modify cysteine residues through S-sulfhydration, thereby altering channel activity. Alternatively, H_2_S might regulate Cx expression via activation of transcription factors or epigenetic modifications, such as DNA methylation (**Figure 2**).

Thus, gasotransmitters form a unique regulatory network that influences Cx function both directly and via complex molecular cascades, modulating intercellular communication in both physiological and pathological contexts. However, their role in Cx regulation during neurotraumas remains poorly understood.

## Role of Gasotransmitters in Connexin Regulation During Neurotrauma

### Nitric oxide

NO affects the structure and function of Cx via S-nitrosylation of cysteine residues, phosphorylation of serine/threonine sites, activation of calcium signaling, ATP release, and regulation of mitochondrial activity.^10^,^11^,^14^,^15^,^16^,^17^,^18^ These mechanisms underlie both neuroprotective and neurotoxic effects in nervous system injuries.

Studies have shown that in astrocytes, NO induces S-nitrosylation of cysteine residues in Cx43 hemichannels, calcium homeostasis modulator 1 channels, and pannexin-1 channels, thereby increasing their permeability. It promotes ATP release and amplifies glutamate-initiated calcium waves.^14^,^70^,^71^ Such molecular mechanisms may mediate neurotoxicity after trauma through enhanced purinergic signaling,^72^ activation of P2X/Y receptors,^73^ excitotoxicity, and calcium overload in neurons,^74^,^75^ and high Cx43 expression levels negatively correlated with clinical severity in patients with TBI.^76^ In *Cx43*/*Cx30* knockout mice, reduced gliosis was observed alongside increased neuropathic pain symptoms after SCI.^77^ It has been reported that a reduction in the protein and mRNA levels of Cx43 contributes to improved neurological recovery after SCI.^78^ Moreover, inhibition of Cx43 hemichannels using a chimeric human-mouse antibody effectively reduced the extent of secondary damage by suppressing gliosis and neuronal death in the injured spinal cord.^8^ Blocking Cx43 channels with the mimetic peptide Peptide5 improved neuronal survival and reduced astrocytic, macrophage, and microglial responses after SCI.^9^ In the peripheral nervous system, inhibition of Cx43 reduced neuropathic pain by downregulating P2X4 receptor expression, as well as tumor necrosis factor alpha and interleukin (IL)-1β in the dorsal root ganglia (**Figure 3A**).^79^

**Figure 3:**
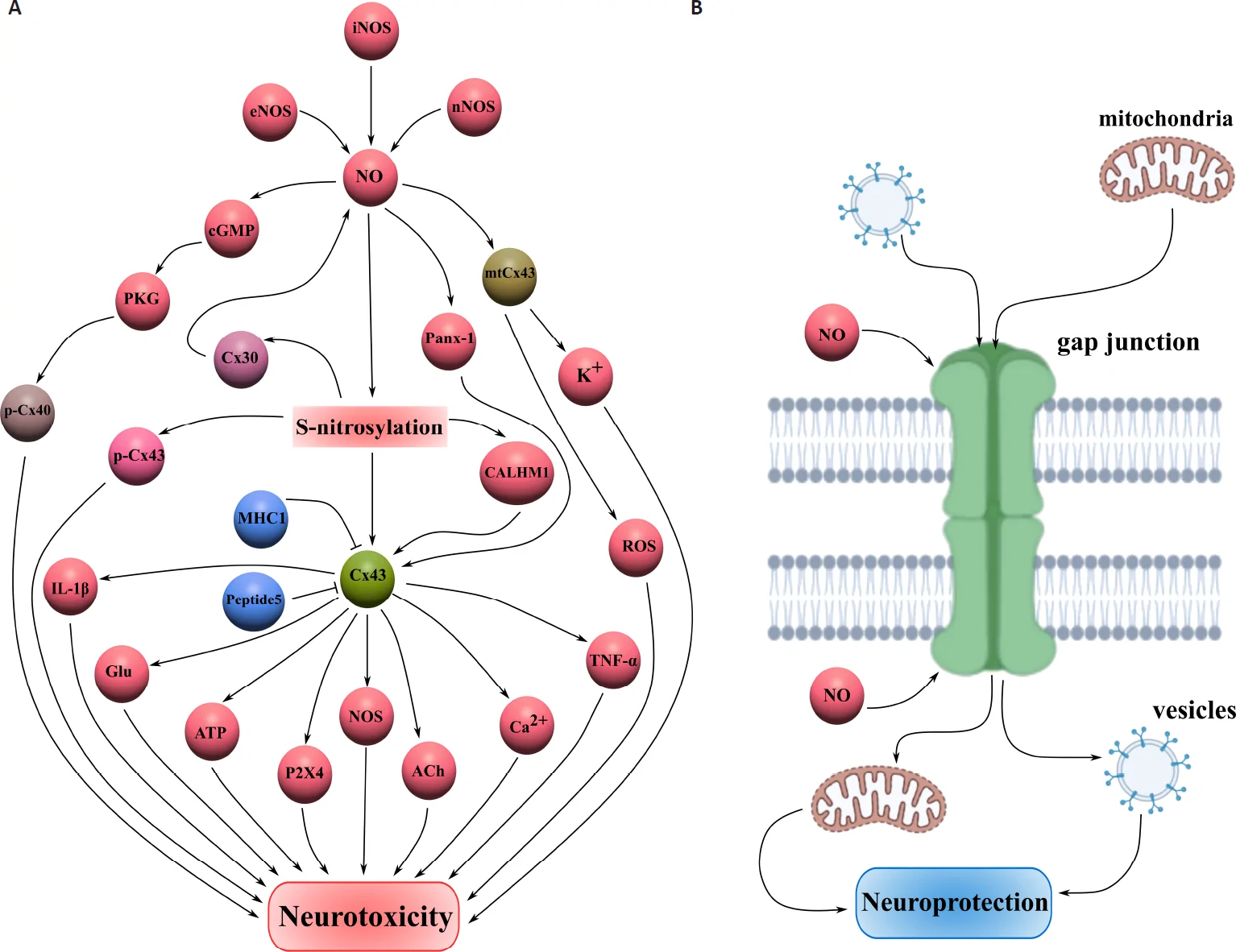
NO-dependent regulation of Cx in neurotrauma. A) Neurotoxic signaling pathways of NO-mediated Cx modulation. (B) Neuroprotective signaling pathways of NO-mediated Cx modulation. Arrows with a sharp end indicate positive regulation; arrows with a blunt end indicate negative regulation. Created with BioRender.com and Inkscape. ACh: Acetylcholine; ATP: adenosine triphosphate; Ca^2+^: calcium ion; CALHM1: calcium homeostasis modulator 1; cGMP: cyclic guanosine monophosphate; Cx: connexin; eNOS: endothelial nitric oxide synthase; Glu: glutamate; IL-1β: interleukin-1 beta; iNOS: inducible nitric oxide synthase; K^+^: potassium ion; MHC1: mouse-human chimeric antibody against Cx43; mtCx43: mitochondrial Cx43; nNOS: neuronal nitric oxide synthase; NO: nitric oxide; P2X2: purinergic receptor P2X2; Panx-1: pannexin-1; p-Cx40: phosphorylated connexin 40; p-Cx43: phosphorylated connexin 43; Peptide5: connexin 43 mimetic peptide 5; PKG: protein kinase G; ROS: reactive oxygen species; TNF-α: tumor necrosis factor alpha.

However, another study reported that astrocytic gap junctions composed of Cx43 reduce neuronal apoptosis in cerebral ischemia models by activating reactive astrocytes, which create a favorable extracellular environment for neurons.^80^ Additionally, Cx43 may serve as a molecular bridge for mitochondrial transfer from astrocytes to injured neurons, enhancing their viability after TBI.^49^ In oxygen-glucose deprivation and reperfusion models, Cx43 overexpression decreased intracellular Ca^2+^ levels, inhibited calcium/calmodulin-dependent protein kinase II/cyclic adenosine monophosphate response element-binding protein signaling and Ephrin-A4 expression, thus protecting astrocytes and promoting neurite regeneration. In middle cerebral artery occlusion, increased Cx43 expression in astrocytes correlated with improved neuronal recovery (**Figure 3B**).^81^ In the spiral ganglion, gap junctions composed of Cx43 in satellite glial cells supported neuronal survival and preserved the electrical excitability of neurons during dendritic degeneration. It confirms the role of Cx43-mediated intercellular communication through gap junctions between satellite glial cells in maintaining the homeostasis of injured neurons and promoting neuronal regeneration.^82^

In a model of metabolic inhibition of cortical astrocytes, it was demonstrated that the number of Cx43 hemichannels increases via mechanisms involving dephosphorylation and S-nitrosylation, where the interaction of NO with intracellular cysteine residues of Cx43 may represent a key mechanism regulating hemichannel permeability.^83^ S-nitrosylation of Cx43 has been reported to correlate with increased levels of serine 368-phosphorylated Cx43 (p-Cx43) and hemichannel opening.^10^ In nervous tissue, p-Cx43 is typically present at low levels and is generally associated with gap junctional functions and the development of pathological processes.^48^ TBI induces a rapid accumulation of p-Cx43 within the first few hours and up to 24 hours post-injury, contributing to the propagation of secondary damage in the brain.^84^,^85^ However, p-Cx43 can exert both neurotoxic effects and neuroprotective actions, including the stimulation of exosome release following TBI (**Figure 3B**).^48^ It has also been shown that NO can oxidize Cx46, specifically targeting its C-terminal domain. Treatment with S-nitrosoglutathione, a NO donor, altered the activation kinetics of Cx46-associated hemichannels.^62^ Overall, various NO donors may enhance S-nitrosylation of Cx46, promoting the activation of associated hemichannels. Nevertheless, Cx46 gap junctions exhibit resistance to NO (**Figure 3A**).^11^ Notably, *Cx46* mRNA levels rapidly increase in degenerating regions following sciatic nerve injury, indicating an important role for this Cx in regenerative processes in the peripheral nervous system.^86^ Expression of Cx46 has also been reported in olfactory ensheathing cells, astrocytes, and Schwann cells.^87^

In microglia activated by lipopolysaccharide, NO produced by iNOS interacts with pannexin-1 channels, enhancing ATP release independently of Cx43 hemichannels.^88^ Panx proteins are recognized homologs of Cx that are also capable of forming transmembrane channels.^89^ Additionally, it has been shown that NO induced by pro-inflammatory cytokines, such as interleukin (IL)-1β and tumor necrosis factor alpha, through iNOS activation, enhances both the expression and function of Cx43. Exogenous NO reproduces this effect, whereas inhibition of iNOS blocks it. Interestingly, Cx43 itself may exert inhibitory effects on iNOS expression (**Figure 3A**).^90^ Cx43 and Panx1 share structural and functional similarities. Both proteins possess four transmembrane domains with similar topology, including cytoplasmic N- and C-termini, two extracellular loops, and one cytoplasmic loop. They form large-pore, low ion-selectivity channels, and are activated by comparable stimuli such as elevated intracellular Ca^2+^, mechanical stress, and changes in membrane potential. Both contribute to overlapping physiological processes, including ATP release and regulation of inflammatory responses. Moreover, Panx1 is capable of modulating the activity of Cx43 hemichannels, suggesting functional crosstalk between these proteins despite their evolutionary divergence.^91^

Microglial activation is accompanied by the opening of Cx43 hemichannels, which promotes the spread of inflammatory responses via inflammasome assembly.^89^ Microglia play a critical role in the CNS following traumatic injury. Early microglial activation after TBI supports the maintenance of neuronal homeostasis. However, prolonged activation leads to neurotoxicity through the release of pro-inflammatory molecules, exacerbating secondary injury processes.^92^ SCI, similar to TBI, triggers rapid microglial activation, which may exhibit dual biological effects depending on the context.^93^ Microglia serve as active producers of NO, a key destructive molecular agent in neuroinflammation.^94^ Its interaction with Cx channels can enhance the release of ATP and ROS,^95^ further aggravating neurotoxic cascades in the context of neurotrauma (**Figure 3A**).

In a model of inflammatory bowel disease, NO produced by glial iNOS induces S-nitrosylation of Cx43, activating hemichannels and releasing ATP, which leads to enteric neuron death (**Figure 3A**).^96^ Several studies have demonstrated a significant relationship between TBI and the structural-functional integrity of the gut, which may acutely respond to traumatic brain damage.^97^,^98^,^99^ The gut-brain axis is a finely tuned pathway involving feedback mechanisms through which neurodegenerative processes affect gastrointestinal function, which in turn negatively influences CNS recovery after injury.^99^ The enteric nervous system is a complex neuronal network located in the gut that regulates gastrointestinal activity. In particular, TBI is associated with peripheral serotonin homeostasis disruption, impairing the innervation of the mucosa by neural waves of the enteric nervous system and triggering a feedback cascade toward the injured CNS.^100^ NO is a key mediator in the enteric nervous system. Various pathological conditions lead to the development of enteric neuropathies due to disturbed Ca^2+^ homeostasis, inducing NO overproduction, resulting in nitrosative stress and the death of neurons and glial cells (**Figure 3A**).^101^

In the model of ischemic preconditioning, NO supports Cx43 phosphorylation, preventing gap junctional coupling and protecting tissue from ischemia. Administration of S-nitroso-N-acetyl-D,L-penicillamine, which spontaneously releases NO in aqueous solutions and is widely used in biology and medicine, reduced cell death through p-Cx43-dependent gap junction uncoupling mechanisms in a myocardial infarction model. The effect was not observed with the use of the non-selective NOS inhibitor L-NG-nitro-arginine methyl ester or a peptide inhibiting the translocation of protein kinase C-ε.^102^ A cardioprotective effect was also observed with nitrate administration, accompanied by increased p-Cx43 levels. However, injection of 2-(4-carboxyphenyl)-4,4,5,5-tetramethylimidazoline-1-oxyl-3-oxide, an NO scavenger, abolished the cardioprotection (**Figure 3B**).^103^ The expression of p-Cx43 and NOS is highly correlated in hypertension models, indicating the critical role of NO in the regulation of this protein.^104^ The tight interaction between Cx and NO is one of the central mechanisms of cellular adaptation during ischemia and reperfusion.^105^ However, in the nervous system, preconditioning combined with pharmacological blockade of gap junctions significantly increases neuronal survival during cerebral ischemia via suppression of Cx43 hemichannel activity, which otherwise mediates astrocytic glutamate release leading to excitotoxicity and oxidative stress (**Figure 3A**).^106^ On the other hand, another study reported that mice with a knockout of the *GJA1* gene, responsible for Cx43 expression, were insensitive to hypoxic preconditioning, whereas wild-type mice exhibited preconditioning-dependent neuroprotection following middle cerebral artery occlusion. It is suggested that preconditioning stabilizes Cx43 by reducing its degradation under ischemic conditions, leading to an increase in Cx43 hemichannels in the plasma membrane, which, through ATP release into the extracellular space, promotes the accumulation of adenosine, a potent neuroprotective agent (**Figure 3B**).^107^

NO also plays a key role in neurovascular coupling via Cx. In astrocytes, NO activates Cx43 hemichannels through S-nitrosylation, enhancing Ca^2+^ influx and ATP release, thereby promoting dilation of parenchymal arterioles.^14^ Neurovascular coupling is mediated by astrocytes, which serve as intermediaries between neurons and microvessels. Ca^2+^-dependent astrocyte signaling is regulated by Cx43 and Cx30, as well as pannexin-1, and participates in neurovascular regulation. NO activates Cx43 and Cx30 hemichannels via S-nitrosylation, which in turn, through Ca^2+^ influx, enhances activation of Ca^2+^-dependent NOS isoforms.^14^ NO, synthesized by astrocytic eNOS and nNOS, mediates vasodilation through S-nitrosylation of calcium homeostasis modulator 1 channels, which leads to the opening of Cx43 hemichannels and subsequent ATP release necessary for vascular signaling. Glutamate-induced astrocyte activation increases intracellular Ca^2+^, NO-dependent opening of calcium homeostasis modulator 1 and Cx43 hemichannels, and purinergic signaling, which together coordinate a synchronized vascular response ensuring adequate blood supply to active neurons (**Figure 3B**).^71^ NO also activates Cx43 hemichannels via S-nitrosylation, increasing the release of acetylcholine, an endothelium-dependent vasodilator.^15^ Furthermore, Cx channels facilitate NO transport into endothelial and smooth muscle cells. It has been shown that HeLa cells lacking Cx are unable to transport NO from the extracellular space into the cytoplasm. In contrast, cells with normal expression of Cx43, Cx40, or Cx37 efficiently transported NO, indicating their critical role in NO-dependent vascular dilation.^108^ S-nitrosylation of Cx43 in myoendothelial junctions is reported to regulate gap junction communication between endothelium and smooth muscle. The process is mediated by eNOS, which nitrosylates Cx43 and enhances intercellular coupling, whereas phenylephrine causes denitrosylation via activation of S-nitrosoglutathione reductase, reducing channel permeability (**Figure 3**).^16^ Excessive vasodilation is believed to play a key role in the pathogenesis of TBI, promoting secondary injury mechanisms, cerebral edema, and intracranial hypertension, while NOS inhibitors may have beneficial effects.^109^ However, endothelial dysfunction, manifested by reduced eNOS expression and increased arginase activity, which depletes the L-arginine substrate necessary for NOS, also occurs during TBI. The molecular-cellular event becomes a major contributor to oxidative stress and cell death in the lesion area.^110^ Additionally, in SCI, inhibition of NO production with Nω-nitro-L-arginine methyl ester led to reduced free radical activity and increased expression of vascular endothelial growth factor.^111^ Importantly, different NOS isoforms may exert opposing effects in SCI. Activation of the eNOS/NO/cGMP signaling pathway has a neuroprotective effect and plays a crucial role in maintaining vascular homeostasis. In contrast, increased iNOS expression is associated with expansion of secondary injury and destabilization of vascular tone regulation mechanisms.^112^ In our previous study, we also showed that iNOS activation in the dorsal root ganglia after sciatic nerve transection correlated with activation of apoptotic signaling and the death of neurons and glial cells.^113^ NO-dependent excessive opening of Cx43 hemichannels may contribute to impaired vascular tone regulation in various types of neurotrauma. The NO/Cx43 signaling pathway can both improve cerebral perfusion during TBI and SCI and contribute to edema, Ca^2+^ overload, and subsequent hyperactivation of constitutive NOS isoforms (**Figure 3A**).

An important member of the Cx family is Cx35, which is the fish ortholog of the mammalian Cx36 and shows a high degree of similarity to it, approximately 90%. Cx36 is widely expressed in the CNS of mammals, in regions such as the hippocampus, cerebellum, and cerebral cortex.^114^ It has been shown in *in vitro* models using HeLa cells expressing Cx35 that NO activates protein kinases G (PKG) and protein kinase A, which phosphorylate serine residues 110, 276, and 289, leading to conformational changes and uncoupling of gap junctions.^115^ Moreover, asymmetric dimethylarginine, a non-selective NOS inhibitor, has been shown to reduce Cx36 expression in neurons under oxygen–glucose deprivation, exerting a neuroprotective effect. It is known that Cx36 may contribute to neuronal and glial cell death during cerebral ischemia.^116^ In a controlled cortical impact model in mice, the administration of mefloquine, a selective gap junction blocker, and the use of transgenic mice incapable of expressing Cx36, led to reduced neuronal death in the damaged area.^117^ Chronic constriction injury of the sciatic nerve resulted in the development of neuropathic pain mediated by altered Cx36 localization due to endoplasmic reticulum stress, forming pathological intercellular connections in the CNS.^118^

In endothelial cells, NO phosphorylates Cx40 via cGMP/protein kinase A signaling, stabilizing its integration into gap junctions and enhancing intercellular communication.^63^,^64^ This NO-dependent mechanism may contribute to improved neurovascular coupling after TBI by reducing hypoxia through vasodilation and increasing the survival of injured neurons and glial cells (**Figure 3B**). However, enhanced oxygenation via Cx40-associated gap junctions might also exacerbate oxidative stress in the injured nervous tissue against a background of reduced antioxidant defenses. For example, Cx40 may contribute to secondary injury in TBI, correlating with increased cerebral oxidative stress (**Figure 3A**).^119^ The administration of ginsenoside Rb1 has been shown to reduce TBI-induced Cx40 expression, exerting a neuroprotective effect.^120^ In a subarachnoid hemorrhage model, NO, through PKG activation, increased Cx40 expression, promoting vasodilation. The effect was mediated by diethylenetriamine/NO, a slow-releasing NO donor with a long half-life. In subarachnoid hemorrhage, reduced levels of Cx40, eNOS, soluble guanylate cyclase, and PKG were observed, contributing to enhanced cerebral vasospasm. However, diethylenetriamine/NO exerted a vasodilatory effect after subarachnoid hemorrhage by activating the cGMP/PKG signaling cascade and increasing Cx40 expression.^121^

Studies have also shown that NO can selectively affect individual Cx isoforms in the vascular system.^122^,^123^ The use of S-nitroso-N-acetyl-D,L-penicillamine in endothelial cells expressing Cx37, Cx40, and Cx43 significantly reduced fluorescent dye transfer between cells via inhibition of Cx37.^122^ NO also plays a key role in regulating Ca^2+^-dependent signaling between vascular cells through selective action on Cx37, contributing to fine-tuned vasomotor activity.^123^ Moreover, Cx37 and Cx40 are co-localized with eNOS in the endothelium and are involved in the regulation of vascular tone. Cx37 plays a more significant role in basal NO release and the response to acetylcholine, whereas the absence of Cx40 reduces Cx37 expression but does not directly affect sensitivity to exogenous NO.^124^ Regulation of vascular function via gap junctions associated with Cx37 may stabilize vascular function after neurotrauma by reducing hypoxia and maintaining normal trophic support. Cx37 has been shown to be expressed in endothelial cells and pericytes at the blood–brain barrier, participating in intercellular signaling, regulating barrier permeability and vascular tone, especially under conditions of inflammation and oxidative stress.^125^ Cx37 expression has also been identified in the spinal cord and dorsal root ganglia. It is assumed that Cx37 plays a key role in the development of these structures and vasculogenesis, and is also of critical importance for maintaining vascular neurohomeostasis and the normal functioning of neurons and glial cells.^126^

In mitochondria, NO regulates Cx43, affecting energy metabolism. S-nitrosylation of cysteine residues in mitochondrial Cx43 (mtCx43) in cardiomyocytes increases mitochondrial membrane permeability to potassium and stimulates ROS production via complex I of the respiratory chain (**Figure 3A**).^127^,^128^ It has been shown that S-nitrosylation of mtCx43 channels using NO donors such as S-nitrosoglutathione and S-nitroso-N-acetyl-D,L-penicillamine increases intracellular K^+^ and ROS levels without affecting Na^+^ levels.^127^ The use of S-nitrosoglutathione significantly increased mitochondrial membrane potential, ATP production, ROS levels, and complex I activity of the electron transport chain through the activation of mtCx43 hemichannels.^128^ Inhibition of Cx43 translocation into mitochondria may lead to cell death.^129^ Expression and phosphorylation of mtCx43 are significantly reduced in cerebral ischemia-reperfusion. However, treatment with carbenoxolone, a gap junction blocker, and diazoxide, a mitochondrial ATP-sensitive potassium channel agonist, not only reduced the damage area but also restored phosphorylated mtCx43 levels, improved mitochondrial morphology, and activated the antioxidant defense system. It is suggested that mtCx43 is a necessary component of neuroprotection via protein kinase C activation and modulation of mitochondrial ATP-sensitive potassium channels during brain injury.^130^ It was also demonstrated that ischemia leads to a reduction in both phosphorylated mtCx43/mtCx43 and phosphorylated phosphoinositide 3-kinase/protein kinase B, which participates in Cx43 phosphorylation. Salvanolic acids reduced astrocyte apoptosis through mtCx43 and phosphoinositide 3-kinase/protein kinase B activation, restoring normal mitochondrial function.^131^

In the Duchenne muscular dystrophy model, S-nitrosylation of Cx43 at cysteine 271 alters hemichannel conformation, causing pathological opening and calcium influx (**Figure 3A**).^17^,^18^ Dystrophin (DP), whose deficiency is observed in Duchenne muscular dystrophy, not only stabilizes muscle fibers but also plays a crucial role in neurons and glial cells, maintaining cell structures, modulating synaptic plasticity, and neurovascular coupling.^132^ It has been reported that DP71 deficiency is a factor in aquaporin-4 (AQP4) depolarization, inducing brain edema and neuroinflammation during cerebral ischemia.^133^ AQP4, in turn, may negatively regulate Cx43 in astrocytes, reducing intercellular communication, suggesting a connection between gap junctions and water channels in the nervous system.^134^ Based on this, it is possible that NO, DP, and Cx form a complex signaling network during neurotrauma, where the imbalance of one component destabilizes the others. For example, DP-dependent disruption of AQP4 polarization may suppress Cx43 activity in astrocytes. Simultaneously, excessive NO-induced S-nitrosylation of Cx43 leads to pathological hemichannel opening, causing massive Ca^2+^ influx and excitotoxicity, exacerbated by DP deficiency, which normally stabilizes membrane complexes and prevents uncontrolled channel activation. As a result, a vicious cycle may form, where the combination of NO-mediated Cx43 modification, DP dysfunction, and AQP4 disruption enhances edema, neuroinflammation, and cell death.

### Carbon monoxide

CO modulates various signaling pathways under both physiological and pathological conditions, including neurotrauma. Due to its unique physicochemical properties, it easily crosses biological membranes and enters cells, where it interacts with various intracellular targets. For example, CO can regulate the activity of different enzymes and transcription factors through mechanisms involving interactions with transition metals or protein carbonylation, causing conformational changes in proteins.^24^,^135^ Additionally, CO can regulate the activity of ion channels, including those associated with Cx, significantly affecting intercellular communication.^135^

Studies have shown that intrathecal administration of carbon monoxide-releasing molecule (CORM)-2 (CORM-2), an effective CO donor, induces antiallodynic and antihyperalgesic effects by reducing Cx43 expression and inhibiting the activity of associated hemichannels and gap junctions in astrocytes following spinal nerve ligation. Notably, CORM-2 also exhibited an opposing effect by decreasing HO-1 expression, which normally suppresses Cx43 production via a CO-dependent mechanism. Conversely, Cx43 overexpression induced by spinal nerve ligation could attenuate the pain-relieving effects of CORM-2 on mechanical and thermal stimuli and enhance the frequency of spontaneous excitatory synaptic signals.^136^ The regulatory role of CORM-2 on Cx has also been demonstrated in oocytes, HeLa, and MCF-7 cells. CORM-2 inhibited Cx46-mediated gap junctions, as well as Cx43 and Cx46 in HeLa and MCF-7 cells. CO is suggested to directly interact with Cx46 through carbonylation mechanisms (**Figure 4**).^19^ However, the use of hemin, an HO-1 inducer, exerted a cytoprotective effect by increasing Cx43 levels in cardiomyocytes in an ischemia-reperfusion model. The cardioprotection was not attributed to activation of the CO/soluble guanylate cyclase/cGMP signaling pathway.^137^

**Figure 4:**
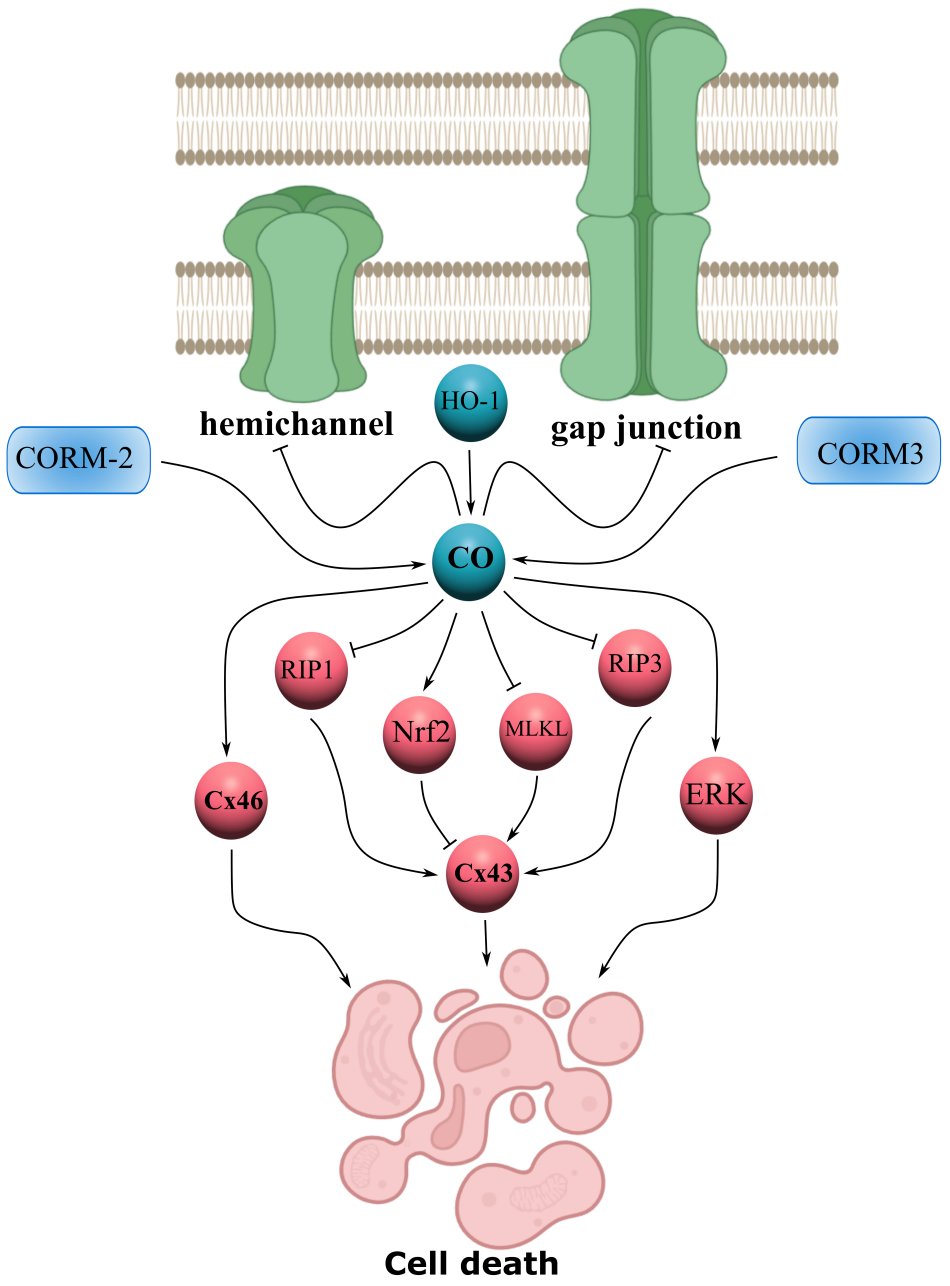
CO-dependent regulation of Cx in neurotrauma. Arrows with a sharp end indicate positive regulation; arrows with a blunt end indicate negative regulation. Created with BioRender.com and Inkscape. CO: Carbon monoxide; CORM: carbon monoxide-releasing molecule; Cx: connexin; ERK: extracellular signal-regulated kinase; HO-1: heme oxygenase-1; MLKL: mixed lineage kinase domain-like protein; Nrf2: nuclear factor erythroid 2-related factor 2; RIP: receptor-interacting protein kinase.

Moreover, the Nrf2/HO-1/CO signaling pathway plays a crucial role in regulating Cx43 expression in the spinal cord under vincristine-induced peripheral neuropathic pain. L-tetrahydroprotoberberine, an alkaloid from the tetrahydroprotoberberine group, mitigated the neurotoxic effects of vincristine by activating Nrf2, which in turn enhanced the HO-1/CO signaling cascade, leading to the inhibition of Cx43 hemichannel activity (**Figure 4**).^67^

In contrast, another study using an intracerebral hemorrhage model demonstrated a neuroprotective effect mediated by increased Cx43 expression, which was necessary for the nuclear translocation of Nrf2 in astrocytes co-cultured with bone marrow-derived mesenchymal stem cells. Transplantation of bone marrow-derived mesenchymal stem cells into the injured brain area reduced astrocyte death through the modulation of Cx43, Nrf2, HO-1, as well as glial fibrillary acidic protein and vimentin.^138^ Overall, Nrf2 is regarded as a transcription factor with powerful neuroprotective properties, activating anti-apoptotic genes and the antioxidant defense system during neuron stress caused by traumatic injury.^139^,^140^,^141^ The modulation of the Nrf2/Cx43 signaling pathway is not unidirectional and involves feedback mechanisms, where Cx43 itself may regulate Nrf2 expression. However, these mechanisms remain poorly understood, particularly in neurons and astrocytes.^142^

The use of CORM-3, another CO donor, led to reduced Cx43 expression and decreased levels of kinases and necroptosis markers receptor-interacting protein kinase 1, receptor-interacting protein kinase 3, and MLKL induced by lipopolysaccharide in an acute lung injury model (**Figure 4**). CO exhibited a pronounced cytoprotective effect through Cx43 modulation by suppressing extracellular signal-regulated kinase-dependent signaling and blocking necroptosis-related cell death.^66^ Furthermore, in a model of cerebral ischemia induced by distal middle cerebral artery occlusion, MLKL was shown to interact with Cx43 at Ser454, resulting in excessive intracellular Ca^2+^ accumulation and enhanced necroptotic signaling. MLKL also inhibited Cx43 ubiquitination, preventing its proteasomal degradation.^143^ Necroptosis is a critically important molecular and cellular event in TBI and SCI.^144^ Unraveling CO/Cx43-dependent mechanisms regulating this type of programmed cell death opens new avenues for understanding the subtle molecular processes involved in neurotrauma and their therapeutic modulation aimed at reducing neurotoxicity.

### Hydrogen sulfide

Studies have shown that H_2_S can regulate platelet aggregation through its interaction with gap junctions. The use of H_2_S-releasing aspirin or sodium hydrosulfide (NaHS) was found to inhibit platelet aggregation by suppressing intercellular communication via gap junctions (**Figure 5**).^20^ Currently, it is known that TBI induces platelet dysfunction, which correlates with poor outcomes.^145^,^146^,^147^ It has also been reported that platelet-rich plasma promotes neuronal regeneration and angiogenesis in SCI.^148^ However, excessive activation of endothelial cells induced by SCI may contribute to heightened platelet reactivity and subsequent thrombus formation.^149^

**Figure 5:**
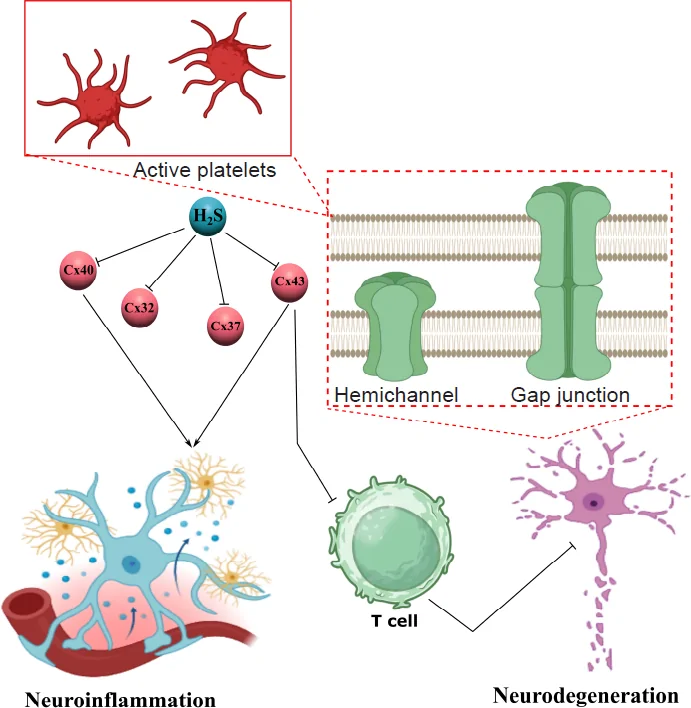
H_2_S-dependent regulation of Cx in neurotrauma. Arrows with a sharp end indicate positive regulation; arrows with a blunt end indicate negative regulation. Created with BioRender.com and Inkscape. Cx: Connexin; H_2_S: hydrogen sulfide.

Cx modulate platelet aggregation by forming gap junctions involved in clot retraction. For example, in mice lacking the *Cx37* gene, platelet aggregation, fibrinogen adhesion, platelet degranulation, and clot retraction were reduced.^150^ Inhibition or knockout of Cx40 in platelets produced similar results.^151^ In addition, some studies have detected the expression of Cx32 and Cx43 in platelets.^152^,^153^ It is also noteworthy that H_2_S levels in neural tissue are maintained at relatively high concentrations due to the activity of cystathionine β-synthase and respond rapidly to neuronal stress induced by trauma.^24^,^69^ Thus, it can be hypothesized that H_2_S may exert its biological effects via Cx-dependent mechanisms of platelet response during neurotrauma.

H_2_S also inhibits neuroinflammation by regulating immune homeostasis and suppressing the expression of Cx40 and Cx43. For instance, administration of NaHS decreased the levels of Cx40 and Cx43, accompanied by reduced expression of IL-2 and IL-6, and a decreased CD4/CD8 ratio, while increasing levels of IL-10 and regulatory T cells (Tregs) (**Figure 5**).^21^ H_2_S is known to inhibit neuroinflammation by activating antioxidant defense systems and suppressing the expression of pro-inflammatory cytokines.^69^ In turn, Tregs exert neuroprotective effects in TBI by limiting inflammation and secondary brain damage. Depletion of Tregs leads to increased infiltration of T cells into the injured brain, heightened reactive astrogliosis, and elevated expression of pro-inflammatory interleukins.^154^,^155^ Similarly, Tregs may promote spinal cord recovery after injury by modulating the activity of macrophages/microglia, oligodendrocytes, astrocytes, and other cells.^156^ Furthermore, Cx43 is reported to be essential for the development of Tregs.^157^ Therefore, a potential H_2_S-dependent neuroprotective mechanism involving Cx in neurotrauma may include inhibition of neuroinflammation and activation of Tregs.

H_2_S can also regulate vascular function by modulating Cx expression and associated gap junctions. For example, in a rat model of ischemia with diabetes, reduced expression of Cx43 and H_2_S in the myocardium was associated with endothelial dysfunction (**Figure 5**). Administration of NaHS and the amylin agonist pramlintide restored Cx43 levels, improved vascular relaxation, and reduced myocardial injury, but these effects were blocked by carbenoxolone, a gap junction inhibitor.^12^ It has also been reported that H_2_S increases Cx40 and Cx43 levels by normalizing nicotinamide adenine dinucleotide phosphate oxidase and protein kinase C-ε activity in the vascular system.^13^

## Gasotransmitter-Associated Trials/Drugs for Modulation of Connexins

To date, convincing preclinical evidence has demonstrated the key role of gasotransmitters in regulating multiple signaling mechanisms associated with neuronal and glial cell survival and death, making them potential candidates for the development of neuroprotective drugs and clinical trials. Their ability to modulate intercellular communication associated with Cx channels, which are considered molecular targets for neuroprotective therapy, appears particularly relevant. For example, the Cx43 hemichannel inhibitor tonabersat (SB-220453) blocked CNS injury-induced neuroinflammation in various models, including ischemia and reperfusion.^158^ The modulator has undergone clinical trials (NCT00534560, NCT00311662, and NCT00332007) evaluating its use for the treatment and prevention of migraine aura. Another noteworthy clinical study (NCT03182413) assessed the effects of combined therapy with THN10, consisting of modafinil and a low dose of flecainide (a glial connexin inhibitor), on cognitive function during sleep deprivation, showing positive effects on psychological well-being.^159^ These findings highlight the need for further development of approaches to modulating Cx levels for neuroprotective therapy, particularly through gasotransmitters. It should be noted that, at present, no gasotransmitter-associated drugs specifically targeting Cx regulation exist.

However, ongoing clinical studies are actively investigating the potential of gasotransmitter-associated drugs for neurotrauma therapy. In particular, one trial (NCT05616910) is evaluating the use of inhaled NO (iNO) in the acute post-traumatic period following TBI. It is hypothesized that iNO may exert neuroprotective effects by improving cerebral blood flow. Another study (NCT04951453) focuses on identifying NO-dependent biomarkers for assessing condition and predicting outcomes in various brain injuries, including TBI. The hypothesis is that NO deficiency, triggered by peroxynitrite (ONOO^−^) formation, initiates a cascade of neurotoxic processes. Additional studies (NCT05871606) are investigating the efficacy of iNO in acute ischemic stroke. Furthermore, a completed study (NCT03260569) analyzed the effects of iNO on respiratory mechanics and gas exchange in TBI patients, while another trial (NCT04988932) is evaluating iNO for severe stroke treatment (**Table 1**). These studies emphasize the potential of iNO as a therapeutic approach for mitigating neurotrauma outcomes and improving clinical prognosis.

**Table 1:** Potential gasotransmitter associated modulators for neuroprotection: drugs and clinical/preclinical trials

Category	Drug/modulator	Status	Effect/application	Reference
NO	iNO	Clinical	Improves cerebral circulation in TBI and ischemic stroke; potential regulation of Cx signaling in vascular/neural response	NCT05616910, NCT05871606, NCT03260569, NCT04988932
	INOMAX®	FDA-approved (NDA #20-845)	Approved for treatment of hypoxic respiratory failure in newborns; candidate for studying NO-dependent Cx regulation in TBI	160,161
	Nitroglycerin, Isosorbide dinitrate, Isosorbide mononitrate	Approved	NO donors for treatment of ischemic heart disease, angina, and heart failure; demonstrated neuroprotective effects; potential Cx modulators	162
	S-nitroso-N-acetylpenicillamine, S-nitrosoglutathione, sodium nitroprusside, diazeniumdiolate, diethylenetriamine/NO, Nω-nitro-L-arginine methyl ester, carboxy-2-phenyl-4,4,5,5-tetramethylimidazoline-1-oxyl-3-oxide	Preclinical	Donors and inhibitors of NO used in preclinical studies; effective in Cx regulation	62 70 90 102 103 111 115 121 122 127
CO	iCO	Clinical (preliminary)	Treatment of pulmonary diseases; limited by toxicity via carboxyhemoglobin formation; potential significance for Cx modulation in neurotrauma	163 NCT00094406, NCT02425579
	CORM-3 (Ru-CO donor)	Preclinical	Reduces pericyte apoptosis, neuroinflammation, and neurological deficits; stimulates neurogenesis after TBI; cytoprotection through blocking Cx expression	66 165
	CORM-A1 (boron-containing CO donor)	Preclinical	Modulates neuronal differentiation, reduces cell death, protects BBB, maintains neuronal homeostasis; probable mechanisms of Cx regulation	166 167
	CORM-2 (Ru-CO donor)	Preclinical	Suppresses neuroinflammation; lipid nanoparticle-modified version shows neuroprotective effect; inhibits Cx-hemichannel activity	67 168 169
	General CORMs	Preclinical	Controlled CO release; various chemical properties and release rates; potential for Cx modulation in neuroprotection	19 136 164
H_2_S	SG1002 (oral H_2_S prodrug)	Clinical	Safety/efficacy in heart failure; increases endogenous H_2_S levels, affects NO bioavailability; potential significance for Cx modulation in neurotrauma	170 NCT01989208
	ATB-346 (H_2_S-releasing)	Clinical (Phase I–II/IIb)	Anti-inflammatory potential; neuroprotective effect in animal models of TBI and SCI; potential candidate for a Cx modulator	171,172 NCT03291418, NCT03978208
	IK-1001 (Sodium sulfide, Na_2_S)	Clinical	For ischemic conditions; reduces neuronal/glial death in TBI; high potential for modulating Cx	173,174 NCT00858936, NCT01007461
	Zofenopril	Approved/clinical	Lipophilic ACE inhibitor, releases H_2_S; cardioprotective effect; stimulates peripheral nerve regeneration in rat sciatic nerve injury model; further studies to assess the impact on Cx	175 176
– (trials with > 3600 myocardial infarction patients)	Sodium thiosulfate	Preclinical	H_2_S donor; protects neurons/glia in traumatic nerve injury; reduces cell death after TBI; potential candidate for a Cx modulator	177 178
	General H_2_S donors (NaHS, Na_2_S)	Preclinical	Fast-releasing H_2_S donors; regulate intercellular communication through Cx; potential neuroprotectors with improved delivery systems	12 20 21

ACE: Antihypertensive; BBB: blood–brain barrier; CO: carbon monoxide; CORM: carbon monoxide releasing molecule; Cx: connexin; FDA: U.S. Food and Drug Administration; H_2_S: hydrogen sulfide; iCO: inhaled carbon monoxide; iNO: inhaled nitric oxide; Na_2_S: sodium sulfide; NaHS: sodium hydrosulfide; NO: nitric oxide; Ru: ruthenium; SCI: spinal cord injury; TBI: traumatic brain injury.

It should be noted that a U.S. Food and Drug Administration-approved iNO drug, INOMAX® (NDA #20-845, Mallinckrodt Pharmaceuticals, Port Allen, LA, USA), is already available for the treatment of hypoxic respiratory failure in newborns. Although studies specifically addressing the role of INOMAX® in Cx regulation have not been conducted, this drug represents a promising candidate for evaluating its potential effects on Cx expression and function, given that iNO has shown beneficial effects in TBI.^160^ However, the rapid inactivation of NO in the bloodstream, with the formation of various metabolites,^161^ limits its use as a targeted neuroprotective drug for Cx modulation in nervous tissue. In this context, it may be worth considering the development of NO-dependent neuroprotectors based on classical drugs capable of releasing NO, such as nitroglycerin, isosorbide dinitrate, and isosorbide mononitrate. These drugs have long been approved and widely used as NO donors for treating ischemic heart disease, angina, and heart failure, and their protective role in nervous tissue has been demonstrated in several studies.^162^ Preclinical NO modulators actively used in laboratory research also show efficacy in regulating Cx, including NO donors such as S-nitroso-N-acetylpenicillamine,^70^,^102^,^122^,^127^ S-nitrosoglutathione,^62^,^127^ sodium nitroprusside, diazeniumdiolate,^115^ diethylenetriamine/NO,^121^ as well as inhibitors of its synthesis (Nω-Nitro-L-arginine methyl ester)^90^,^102^,^111^ and NO scavengers such as carboxy-2-phenyl-4,4,5,5-tetramethylimidazoline-1-oxyl-3-oxide^103^ (**Table 1**).

Considering another gasotransmitter, CO, as a potential neuroprotective Cx modulator, it should be noted that no CO-based drugs have yet been approved. Clinical trials with CO are scarce, preliminary, and unrelated to assessing its safety and efficacy in neurotrauma. For example, clinical trials (NCT00094406 and NCT02425579) investigated inhaled CO for treating pulmonary diseases. The main limitation of inhaled CO therapy is its potential toxicity, as CO readily binds hemoglobin to form carboxyhemoglobin, impairing oxygen transport.^163^ It presents a major barrier for systemic use of inhaled CO due to the risk of dosing errors. A promising solution lies in the development of CORMs, which ensure controlled CO release without a dangerous increase in carboxyhemoglobin concentration. Currently, an entire line of CORMs exists, with varying chemical properties and release rates,^164^ some of which have demonstrated neuroprotective efficacy in preclinical studies. For example, CORM-3, a ruthenium (Ru)–CO donor, reduced pericyte apoptosis, neuroinflammation, and neurological deficits while stimulating neurogenesis in TBI models.^165^ In addition, it was found that CORM-3 can modulate the expression of Cx43, thereby inhibiting cell death.^66^ The water-soluble boron-based donor CORM-A1 positively modulated neuronal differentiation, reduced cell death,^166^ protected the blood-brain barrier, and maintained neuronal homeostasis.^167^ Additionally, the classic lipophilic Ru–CO donor CORM-2 suppressed neuroinflammation by inhibiting microglial activation,^168^ while its solid lipid nanoparticle formulation showed beneficial effects in SCI therapy (**Table 1**).^169^ It should be noted that CORM-2 may exert a neuroprotective effect by inhibiting Cx43 hemichannel activity.^67^ Such a wide range of biological effects may involve modulation of intercellular communication via Cx, given that preclinical studies have already demonstrated CO-dependent Cx regulation,^19^,^136^ which could eventually lead to CO-associated neuroprotective drugs specifically targeting Cx.

H_2_S donors are also of interest, as their use is now transitioning from preclinical to clinical stages. For example, the safety and efficacy of the oral H_2_S-associated prodrug SG1002 are being evaluated in patients with heart failure (NCT01989208). It was shown to increase endogenous H_2_S levels and influence NO bioavailability in the blood.^170^ A series of phase I–II/IIb clinical trials (NCT03291418, and NCT03978208) of ATB-346, an H_2_S-releasing drug, demonstrated its anti-inflammatory potential. ATB-346 has also shown neuroprotective effects in several animal studies of TBI^171^ and SCI.^172^ Trials have also been conducted on IK-1001, a sodium sulfide (Na_2_S) formulation, for treating various ischemic conditions (NCT00858936, and NCT01007461). In our previous research, we demonstrated that Na_2_S administration significantly reduced neuronal and glial cell death induced by TBI by inhibiting the pro-apoptotic protein p53. The effect was also observed in an axotomy model (complete axon transection) and was abolished by aminooxyacetic acid, a classical CBS inhibitor.^173^ We also found that Na_2_S injections reduced iNOS and amyloid precursor protein expression in neurons and glial cells following TBI and axotomy, while aminooxyacetic acid administration had the opposite effect.^174^ Like NaHS, Na_2_S is a fast H_2_S donor that rapidly dissociates to form HS^−^/H_2_S upon administration, quickly reaching biologically active concentrations. Several studies have shown that NaHS can regulate intercellular communication via mechanisms affecting Cx gap junctions.^12^,^20^,^21^ Another clinically relevant compound is zofenopril, a lipophilic angiotensin-converting enzyme inhibitor containing a free thiol group capable of releasing H_2_S, thereby exerting additional biological effects. Zofenopril has undergone clinical trials involving more than 3600 patients worldwide, demonstrating significant cardioprotective effects in myocardial infarction.^175^ It also promoted peripheral nerve regeneration in an experimental sciatic nerve injury model in rats.^176^ In addition, the classical drug sodium thiosulfate, which acts as an H_2_S donor, has been shown to protect neurons and glial cells in various traumatic injuries of nervous tissue.^177^ For example, we demonstrated that sodium thiosulfate effectively reduced cell death, particularly apoptosis, following TBI (**Table 1**).^178^ Thus, H_2_S donors may be considered promising candidates for developing neuroprotective agents capable of modulating Cx activity. However, their practical application will require clinical studies and likely the development of delivery systems ensuring selective targeting of nervous tissue injury sites and more precise molecular modulation.

## Limitations

In designing this study, the available evidence was carefully considered, though certain limitations had to be acknowledged. The data were conceptualized according to the molecular mechanisms by which gasotransmitters act on Cx, including S-nitrosylation, phosphorylation, calcium signaling, regulation of hemichannels and gap junctions, among others. Due to the lack of studies directly examining the effects of gasotransmitters on Cx in the context of neurotrauma, findings from other pathological models were extrapolated to TBI, SCI, and peripheral neurotrauma, taking into account shared pathological processes. A qualitative synthesis was performed, as comprehensive quantitative data were often unavailable.

In this regard, the limitations of the present review are primarily due to the lack of studies directly investigating the role of gasotransmitters in the modulation of Cx expression and function in neurotrauma. Consequently, extrapolation of results from other pathological models introduces potential biases, as these models may not fully reflect the unique pathophysiological processes characteristic of injuries to the central and peripheral nervous systems. Moreover, the heterogeneity of the studies considered including differences in experimental models, protocols for the administration of gasotransmitter donors, and inhibitors of enzymes responsible for gasotransmitter synthesis, did not allow for a reliable quantitative meta-analysis and necessitated an assessment of the data through qualitative synthesis. In addition, the lack of comprehensive data on the mechanisms of interaction between gasotransmitters and Cx limits the fundamental coherence of our understanding of these processes in neurotrauma.

## Conclusion

NO, CO, and H_2_S play an important role in the regulation of Cx under both physiological and pathological conditions. However, there is a fundamental knowledge gap regarding the mechanisms of gasotransmitter-dependent regulation of Cx in injuries to the CNS and peripheral nervous system. In the absence of selective neuroprotective agents, this may represent a critical oversight that must be addressed through future research in this field.

At present, most attention is focused on the role of NO in regulating the expression of Cx and the associated hemichannels and gap junctions, while the involvement of CO and H_2_S in these processes remains largely unexplored.

This review provides a detailed analysis of the impact of gasotransmitters on the pathogenesis of neurotrauma, primarily through their ability to modulate Cx activity. Particular attention is given to the dual role of gasotransmitters, which, depending on the context, may exert both neuroprotective and neurotoxic effects mediated via Cx hemichannels and gap junctions. These processes are shown to be reflected in a wide spectrum of pathophysiological and molecular-biochemical changes associated with neurotrauma, including S-nitrosylation, carbonylation, and S-sulfhydration of connexins, disruption of calcium homeostasis, release of ATP and reactive oxygen species, activation of microglia and the development of neuroinflammation, as well as mitochondrial dysfunction and intercellular mitochondrial transfer. In a gasotransmitter-mediated context, Cx-dependent regulation of neurovascular coupling and vascular tone, interaction with the gut–brain axis and the enteric nervous system, modulation of immune homeostasis through Tregs, and participation in platelet aggregation are also considered. Finally, the review discusses prospects for the development of gasotransmitter-associated therapeutic strategies aimed at the selective regulation of Cx in TBI and SCI.

This study differs from other reviews in that it systematically integrates data on all gasotransmitters in the context of Cx regulation, specifically in neurotrauma, emphasizing their post-translational modifications and signaling pathways, in contrast to existing works that either focus on individual gasotransmitters or on the general aspects of gasotransmitters in CNS and peripheral nervous system injury without highlighting Cx. To date, the investigation of gasotransmitters in Cx regulation during neurotrauma remains underdeveloped, with a predominance of preclinical models and a lack of comprehensive clinical data, which opens promising avenues for future research aimed at the development of targeted therapies.

Based on the available evidence, it can be concluded that NO, CO, and H_2_S may influence Cx through direct post-translational modifications such as S-nitrosylation, carbonylation, and S-sulfhydration, respectively, as well as via complex signaling pathways that regulate Cx expression, trafficking, and channel gating.

Overall, the gasotransmitter-Cx axis represents a promising therapeutic target for mitigating secondary damage following neurotrauma. A deeper understanding of the molecular mechanisms underlying these interactions will aid in the development of novel strategies aimed at modulating intercellular communication, reducing neuroinflammation, and improving outcomes in traumatic injuries of the nervous system.

**Facts**

1. Gasotransmitters directly modulate connexin function through post-translational modifications and complex signaling pathways.
2. Nitric oxide exerts both neuroprotective and neurotoxic effects via S-nitrosylation, phosphorylation, and regulation of connexin channels.
3. Carbon monoxide demonstrates a pronounced neuroprotective effect by reducing the expression of connexins 43 and 46, inhibiting their gap junctions, and activating the transcription factor nuclear factor erythroid 2-related factor 2, which suppresses connexin 43.
4. Hydrogen sulfide exerts antioxidant and anti-inflammatory effects by modulating connexins 43 and 40, influencing platelet aggregation and vascular dysfunction.
5. Connexins act as molecular hubs that integrate gasotransmitter-dependent signals in response to central and peripheral nervous system injuries.

**Open Questions**

1. What are the key molecular mechanisms of gasotransmitter-dependent regulation of connexins in neurotrauma?
2. Which factors determine isoform-specific effects of connexins in response to gasotransmitter modulation during nervous system injury?
3. What concentration ranges and temporal parameters underlie the transition of gasotransmitters from neuroprotection to neurotoxicity?
4. What conceptual principle should guide the development of selective pharmacological modulators of connexins associated with gasotransmitters?
5. What role do interactions between gasotransmitters themselves play in the integration of connexin-regulated signa ling cascades during neurotrauma?

## Data Availability

*All data relevant to the study are included in the article or uploaded as additional files.*
